# Myocardial death and dysfunction after ischemia-reperfusion injury require CaMKIIδ oxidation

**DOI:** 10.1038/s41598-019-45743-6

**Published:** 2019-06-26

**Authors:** Yuejin Wu, Qinchuan Wang, Ning Feng, Jonathan M. Granger, Mark E. Anderson

**Affiliations:** Department of Medicine, Johns Hopkins Medical Institute, Baltimore, MD 21205 USA

**Keywords:** Permeation and transport, Myocardial infarction, Mechanisms of disease, Endocytosis, Translational research

## Abstract

Reactive oxygen species (ROS) contribute to myocardial death during ischemia-reperfusion (I/R) injury, but detailed knowledge of molecular pathways connecting ROS to cardiac injury is lacking. Activation of the Ca^2+^/calmodulin-dependent protein kinase II (CaMKIIδ) is implicated in myocardial death, and CaMKII can be activated by ROS (ox-CaMKII) through oxidation of regulatory domain methionines (Met281/282). We examined I/R injury in mice where CaMKIIδ was made resistant to ROS activation by knock-in replacement of regulatory domain methionines with valines (MMVV). We found reduced myocardial death, and improved left ventricular function 24 hours after I/R injury in MMVV *in vivo* and *in vitro* compared to WT controls. Loss of ATP sensitive K^+^ channel (KATP) current contributes to I/R injury, and CaMKII promotes sequestration of KATP from myocardial cell membranes. KATP current density was significantly reduced by H_2_O_2_ in WT ventricular myocytes, but not in MMVV, showing ox-CaMKII decreases KATP availability. Taken together, these findings support a view that ox-CaMKII and KATP are components of a signaling axis promoting I/R injury by ROS.

## Introduction

Myocardial ischemia is a major public health problem. Modern therapies have improved the prognosis of myocardial ischemia, allowing for reperfusion of occluded coronary arteries^1–3^. Unfortunately, myocardial reperfusion can trigger a complex, and incompletely understood, cascade of events leading to cell death. Increased reactive oxygen species (ROS) are generated by ischemia/reperfusion (I/R), and are thought to contribute to myocardial injury and death^4,5^. However, the pathways activated by ROS, and potentially contributing to I/R injury remain largely unknown. Furthermore, untargeted anti-oxidant therapies have not proven successful^6^, suggesting either that ROS does not play a major role promoting I/R injury, or, alternatively, that more concise targeting of ROS mediated events will be required to achieve a therapeutic result. Improved understanding of ROS dependent pathways activated in I/R injury is an important goal for this field.

The multifunctional Ca^2+^ and calmodulin dependent protein kinase II (CaMKII) is abundant in heart, and is activated by ROS^7–9^. CaMKII is initially activated by binding to calcified calmodulin, but oxidation of regulatory domain methionines, at positions 281 and 282, contributes to persistent calmodulin-autonomous CaMKII activity by preventing autoinhibition^10^. Excessive CaMKII activity is implicated in myocardial death, including in I/R injury^11,12^, suggesting that oxidized CaMKII (ox-CaMKII) could be an important, but previously unexplored, pathological signal in I/R injury. We performed I/R surgery on a knock-in mouse model of ROS resistant CaMKIIδ, the predominant myocardial isoform, where methionines 281/282 were replaced with valines (MMVV)^13,14^, and discovered that MMVV hearts were protected from myocardial death and dysfunction, consistent with the hypothesis that ox-CaMKII is an important step for ROS transduction in I/R injury.

The ATP sensitive K^+^ channel (KATP) is a nutrient sensing protein complex, and increasing KATP current (I_KATP_) contributes to myocardial protection during I/R injury^15,16^. CaMKII can reduce I_KATP_, at least in part, by augmenting sequestration of KATP from the sarcolemma to a cytoplasmic compartment^17,18^. Based on these associations, we hypothesized that ox-CaMKII contributed to I/R injury by reducing I_KATP_. In support of this idea, we found that I_KATP_ recorded from ventricular myocytes isolated from wild type (WT) mice was significantly reduced by application of H_2_O_2_, but that I_KATP_ in ventricular myocytes isolated from MMVV mice was unaffected by H_2_O_2_. Isolated MMVV ventricular myocytes were significantly resistant to death from simulated I/R and from H_2_O_2_ treatment compared to WT ventricular myocytes. In contrast, viability of WT ventricular myocytes after simulated I/R or H_2_O_2_ addition was enhanced by addition of pinacidil, a KATP opener, while MMVV ventricular myocytes were not advantaged, suggesting that loss of ox-CaMKII in MMVV hearts was protective in large part because of greater I_KATP_ in the face of I/R or H_2_O_2_ treatment. We and others previously identified threonine 224 on Kir6.2, the KATP pore forming subunit, as a site where CaMKII catalyzed phosphorylation promotes KATP trafficking away from the sarcolemma, causing reduced I_KATP_, and augmenting myocardial injury and death after I/R injury^18,19^. We developed a new knock-in mouse where Kir6.2 threonine 224 was replaced by alanine (T224A) to explore the potential for this mutation to protect against ox-CaMKII mediated myocardial injury. I_KATP_ in T224A ventricular myocytes was resistant to decreases after H_2_O_2_ treatment, as predicted, but unexpectedly I_KATP_ was reduced at baseline compared to WT and MMVV ventricular myocytes, and viability of T224A ventricular myocytes exposed to simulated I/R was reduced. Collectively, our findings support a view that CaMKII is a pathological ROS sensor, and ox-CaMKII reduces I_KATP_ contributing to myocardial death and dysfunction after I/R injury. It is likely that ox-CaMKII affects KATP by actions at more than one site.

## Results

### Ox-CaMKII is essential for myocardial I/R injury ***in vivo*** and ***in vitro***

Excessive activation of CaMKII contributes to I/R injury in animal models^20–22^, but the upstream pathways responsible for CaMKII activation in I/R are unknown. Increased oxidant stress follows myocardial reperfusion^4^, so we hypothesized that ox-CaMKII^7^ is important for adverse outcomes in response to I/R. Ox-CaMKII can contribute to cardiovascular^13,14^, pulmonary^23^, and other^24^ diseases linked to excessive ROS. We used mice, developed in our laboratory^13,14^, where knock-in replacement of oxidizable regulatory domain methionines (281/282) with valines (MMVV) in CaMKIIδ, the predominant myocardial CaMKII isoform, prevents ox-CaMKII formation^13,14^.

**Figure 1:**
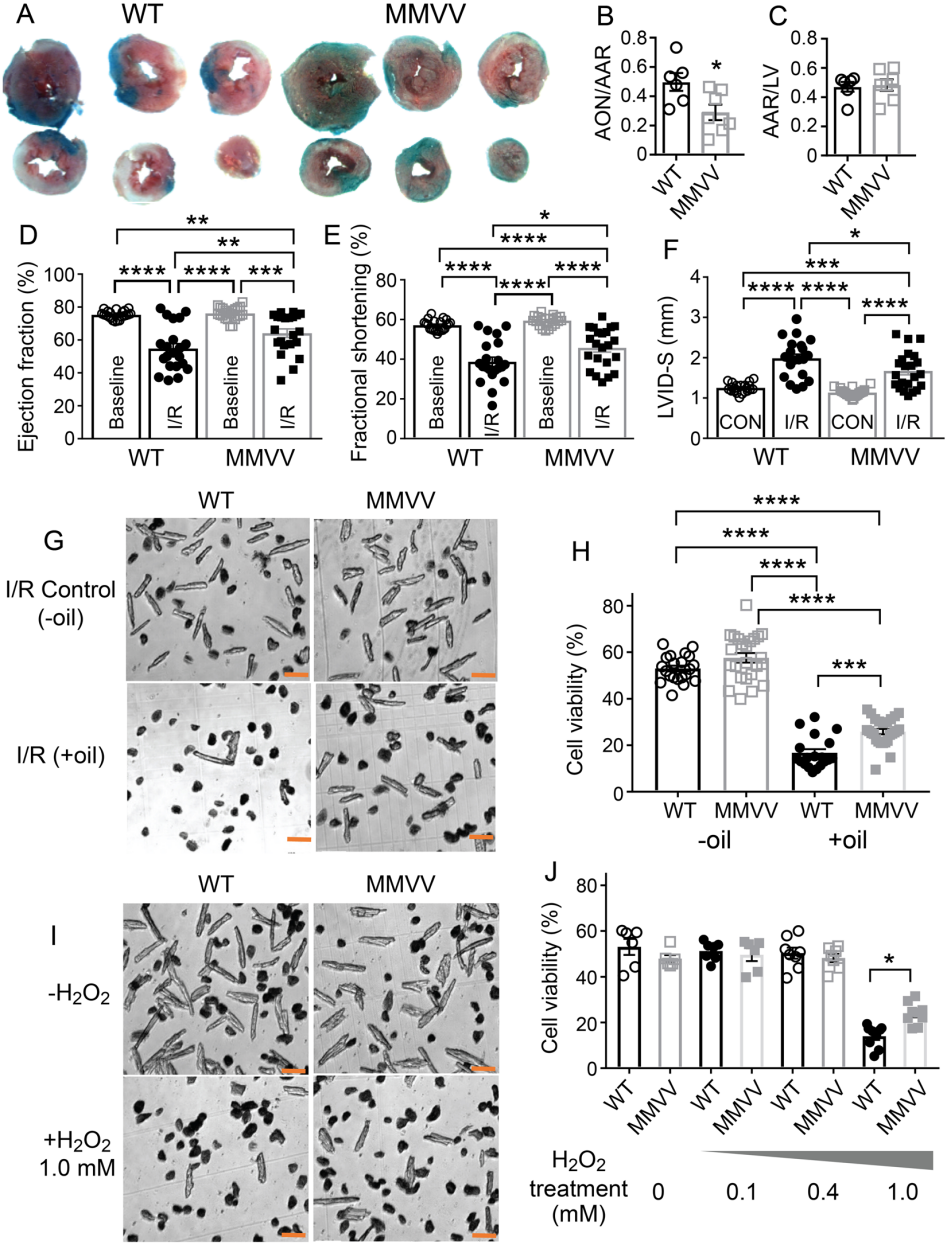
MMVV mice are protected against I/R injury. (**A**) Representative images of transverse cardiac sections from WT (left) and MMVV (right) mice after I/R surgery. Area at risk (AAR) is the sum of red and white areas, and the area of necrosis (AON) is white. The blue area is outside of the AAR. (**B**) Summary data for AON/AAR. The AON/AAR was significantly reduced in MMVV hearts (n = 7) compared to their littermate WT hearts (n = 6). *p < 0.05 unpaired Student’s *t*-test. (**C**) Summary data for AAR measured over the left ventricle (LV). (**D**) LV ejection fractions (**E**) LV fractional shortening (**F**) LVID-S (LV internal diameter in systole) before and after I/R surgery in MMVV (n = 22) and WT (n = 21) mice. One way ANOVA (P < 0.0001). Tukey’s multiple comparisons test compared each group, as indicated by brackets *p < 0.05, **p < 0.01, ***p < 0.001, ****p < 0.0001. (**G**) Representative images of ventricular myocytes isolated from MMVV (right) and littermate WT (left) hearts under I/R control conditions with all experimental steps except addition of an oil layer (−oil, upper) and simulated cellular I/R (+oil, lower). Scale bar is 100 µm. (**H**) Summary data for cell viability expressed as the percentage of live cells. Each point was a summary of 7–10 fields of cell counting (~50–100 cells in each field). Cells from 9 WT mice and 10 MMVV mice. One way ANOVA (P < 0.0001). Tukey’s multiple comparisons test compared each group, as indicated by brackets *p < 0.05, **p < 0.01, ***p < 0.001, ****p < 0.0001. (**I**) Representative images of ventricular myocytes isolated from MMVV (right) and littermate WT (left) hearts under control conditions (−H_2_O_2_, upper) and after addition of 1 mM H_2_O_2_ (+H_2_O_2_, lower). The scale bar is 100 µm. (**J**) Summary data for cell viability based on percentage of live cells, under different concentrations of H_2_O_2_. Cells are from 2 mice from each group. Data were analyzed with a one way ANOVA test (P < 0.0001). Sidak’s multiple comparisons test was used for comparisons between groups. All comparisons between the 1 mM H_2_O_2_ and other groups were significant (P < 0.0001). Significantly more MMVV compared to WT ventricular myocytes were viable after 1 mM H_2_O_2_ (*p < 0.05).

As a first step, we measured area at risk, necrotic, and viable tissue in left ventricular slices 24 hours after I/R surgery. MMVV hearts exhibited less myocardial death compared to WT controls after I/R injury (Fig. 1A,B), despite similar area at risk (Fig. 1C). Twenty-four hours after I/R surgery MMVV mice showed significantly improved left ventricular ejection fractions and fractional shortening, and reduced left ventricular dilation compared to WT mice (Fig. 1D–F, Supplementary Fig. 1A,B). In contrast, MMVV and WT mice had similar echocardiographic measurements at baseline, prior to surgery (Fig. 1D–F, Supplementary Fig. 1A–C). We interpreted these data to support the hypothesis that ox-CaMKII was an upstream signal promoting I/R injury by increasing myocardial death, and leading to worsened myocardial function. Although CaMKIIδ is highly represented in myocardium^25^, it is also expressed in other cells and tissues, raising the possibility that the benefits of the MMVV mutation in I/R injury could arise outside of myocardium. In order to focus on the presumed role of ox-CaMKII in myocardium, we challenged isolated ventricular myocytes using a validated *in vitro* I/R model^26^, by imposing hypoxia with a gas permeability resistant lipid layer followed by removal of the lipid layer and reoxygenation. At baseline, in the absence of I/R conditions, MMVV and WT ventricular myocyte isolation yielded a similar percentage of viable cells (Fig. 1G,H). We found that, similar to our *in vivo* results, isolated ventricular myocytes from MMVV mice were relatively resistant to I/R injury compared to cardiomyocytes isolated from WT mice (Fig. 1G,H). Finally, we measured viability of isolated ventricular myocytes in the presence of increasing concentrations of H_2_O_2_. Under our experimental conditions (see Methods), both MMVV and WT ventricular myocytes had similar viability at baseline, and sustained similar viability after exposure to lower concentrations (0.1 and 0.4 mM) of H_2_O_2_ (Fig. 1I,J). However, MMVV myocytes were protected against death after exposure to 1 mM H_2_O_2_ compared to WT (Fig. 1I,J) to a similar extent as was measured in response to *in vitro* simulated I/R (Fig. 1H). Taken together, the data up to this point were consistent with a model where ROS contributed to myocardial death and dysfunction by a pathway involving ox-CaMKIIδ.

### KATP current is decreased by ox-CaMKII

**Figure 2:**
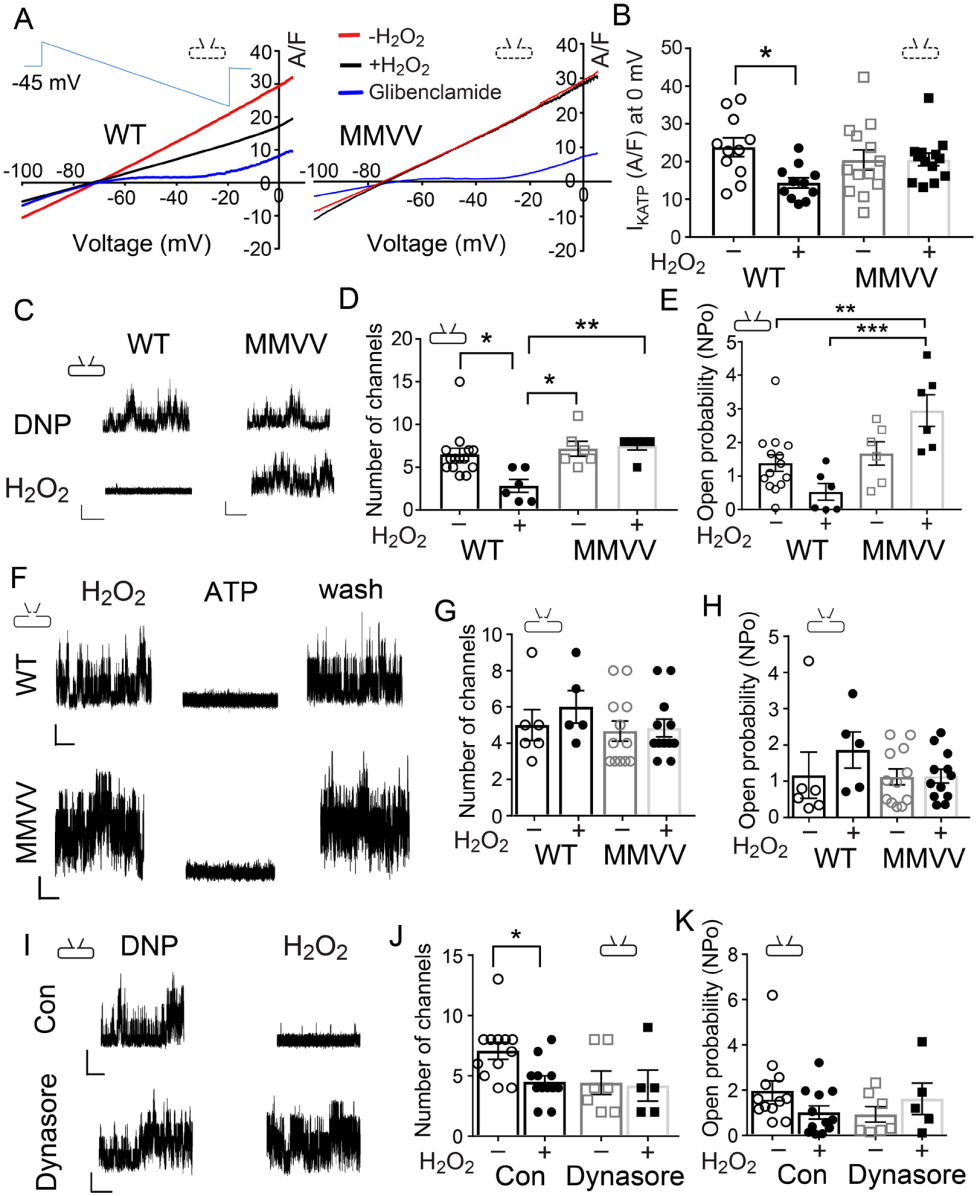
MMVV ventricular myocytes are resistant to H_2_O_2_-induced reduction in I_KATP_. (**A**) Representative traces of I_KATP_ in response to a ramp command pulse (inset) from ventricular myocytes isolated from WT and MMVV mice in the presence of pinacidil and DNP with (black line) and without (red line) H_2_O_2_. Glibenclamide was added to eliminate I_KATP_ (blue line). Inset schematics indicate patch clamp configurations used to obtain each data set, here and throughout. (**B**) I_KATP_ recorded at 0 mV, with and without H_2_O_2_, from ventricular myocytes isolated from WT (n = 11 cells, 2 mice) and MMVV (n = 13 cells, 3 mice) mice. (**C**) Representative traces of KATP channel currents recorded from ventricular myocytes in cell attached mode in the presence of the DNP with and without H_2_O_2_. The vertical scale bar 5 pA, and the horizontal scale bar 2 s (same scale for panel F and I). (**D**) Number of KATP channels opening in each membrane patch under conditions with and without H_2_O_2_. (**E**) KATP channel open probability (NPo) calculated from the membrane patches analyzed in (**D**). WT (6–13 cells, 2 mice), MMVV (6 cells, 2 mice). (**F**) Representative traces of KATP channel recording from excised cell membrane patches in inside-out mode at −60 mV membrane potential and H_2_O_2_ treatment, ATP, and after washout. (**G**) Number of KATP channels opening in each membrane patch under conditions with and without H_2_O_2_. (**H**) KATP NPo calculated from the membrane patches analyzed in (**G**), 5–6 cells from 3 WT mice and 12 cells from 3 MMVV mice. (**I**) Representative KATP channel currents recorded from WT isolated ventricular myocytes in cell attached mode in the presence of DNP with and without H_2_O_2_. The lower panel shows cells pretreated with Dynasore. (**J**) Number of KATP channels opening in each membrane patch under conditions with and without H_2_O_2_ and with and without pretreatment of Dynasore. (**K**) KATP NPo calculated from the membrane patches analyzed in (**J**), 5–12 cells in each group, 3 WT mice. One way ANOVA and Tukey’s multiple comparisons test were used for comparing each group in all the bar graph panels,*p < 0.05, **p < 0.01, ***p < 0.001.

CaMKII reduces cell membrane expression of KATP channels in cardiomyocytes^17,18^, and preventing loss of KATP channels has been proposed as a mechanism for the beneficial actions of CaMKII inhibition in I/R injury^17,27^. Based on these concepts, we asked if ox-CaMKII could decrease I_KATP_ density in ventricular myocytes. We found that I_KATP_ density was reduced by H_2_O_2_ (0.4 mM) in cardiomyocytes isolated from WT (Fig. 2A left panel and Fig. 2B left two bars), but not from MMVV mice (Fig. 2A right panel and Fig. 2B right two bars). We elicited I_KATP_ using a ramp voltage command (see methods, Supplementary Fig. 2 and Fig. 2A inset), and applied pinacidil (0.1 mM) and 2, 4 dinitrophenol (DNP, 0.1 mM) to maximize baseline I_KATP_ (red lines in Fig. 2A for both WT and MMVV representative traces)^28^, and glibenclamide (3–6 µM) to eliminate I_KATP_ (blue lines in Fig. 2A for both WT and MMVV representative traces)^29^. We found that pretreatment of H_2_O_2_ significantly reduced I_KATP_ density in ventricular myocytes isolated from WT mice (black line in the left panel of Fig. 2A and summary data in Fig. 2B), but that H_2_O_2_ application had almost no effect on I_KATP_ density in MMVV ventricular myocytes (black line in the right panel of Fig. 2A and summary data in Fig. 2B). Data acquired under these experimental conditions showed that increased oxidant stress reduced I_KATP_ and that ox-CaMKII was essential for this action.

We next asked if KATP channel opening probability (Po) was different between WT and MMVV cardiomyocytes. On cell-attached mode voltage clamp recordings showed similar Po and KATP channel density at baseline in ventricular myocytes isolated from WT and MMVV mice (Fig. 2C–E). H_2_O_2_ (1 mM) significantly reduced the number of available KATP channels in WT myocyte cell membranes (Fig. 2C,D), however changes in Po after H_2_O_2_ treatment were not significant in WT membrane patches (P = 0.23, Fig. 2E). In contrast, H_2_O_2_ did not reduce KATP density nor Po in MMVV ventricular myocyte sarcolemma patches (Fig. 2C–E). We repeated these studies in excised, inside out, cell membrane patches. In contrast to our findings in whole cell mode perforated patch clamp configuration (Fig. 2A,B) and single channel cell-attached mode recordings (Fig. 2C,D), where cytoplasmic contents were preserved, the KATP channel density and Po were not changed by H_2_O_2_ treatment in excised WT and MMVV ventricular myocyte membrane patches (Fig. 2F–H). This lack of difference in KATP currents in the excised membrane patch recordings before and after H_2_O_2_ suggested that KATP channels had similar intrinsic responses to H_2_O_2_, and that ox-CaMKII actions on I_KATP_ in WT ventricular myocytes arose from a cellular function that was lost during membrane patch excision. We next tested the hypothesis that H_2_O_2_ treatment reduced sarcolemmal KATP channels by endocytosis, using Dynasore, an endocytosis inhibitor. Dynasore (10 μM) eliminated loss of KATP channels by H_2_O_2_ in WT myocytes (Fig. 2I), without affecting KATP channel Po (Fig. 2K). These findings support the hypothesis that ox-CaMKII contributes to reduced I_KATP_ by augmenting KATP channel endocytosis.

### Knock-in mice lacking Kir6.2 T224

**Figure 3:**
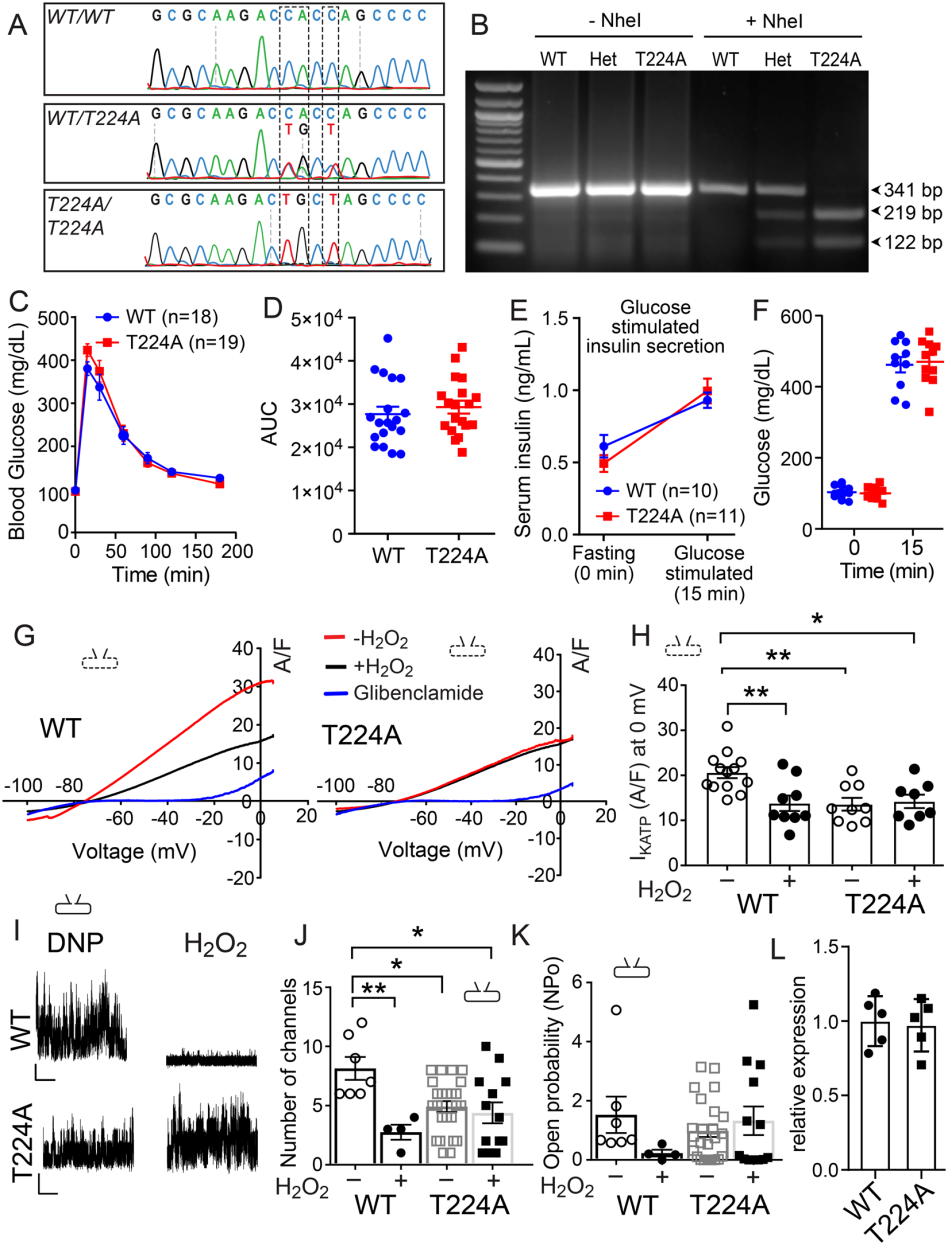
Normal exercise capacity, glucose tolerance, and glucose-induced insulin secretion, but reduced and H_2_O_2_-resistant I_KATP_ in CaMKII resistant KIR 6.2 T224A mice. (**A**) Sanger sequencing results of PCR products amplified from genomic DNA of wildtype (*WT/WT*), heterozygous (*WT/T224A*) and homozygous mutant mice (*T224A/T224A*). (**B**) DNA gel electrophoresis of PCR products before and after NheI digestion. (**C**) *WT* and *T224A* mice were fasted for 16 hours (overnight) and then injected intraperitoneally with 2 g/kg glucose to assess glucose tolerance. No significant difference was found between the *WT* and *T224A* mice. (**D**) Area under the curve (AUC) of the measurements in (**C**). (**E**) Serum insulin concentrations were not significantly different between *WT* and *T224A* mice either after 16-hour fasting or 15 minutes after 2 g/kg glucose injection. (**F**) No difference between genotypes in blood glucose concentrations measured during glucose‐induced insulin secretion. (**G**) Representative I_KATP_ currents recorded from ventricular myocytes isolated from WT and T224A mice using the same protocol and conditions as shown in Fig. 2A. (**H**) I_KATP_ recorded at 0 mV, with and without H_2_O_2_, from ventricular myocytes isolated from WT (n = 9–13 cells, 3 mice) and T224A (n = 8 cells, 3 mice) mice. One way ANOVA and Tukey’s multiple comparisons test were used for comparisons between groups as marked by brackets (*p < 0.05, **p < 0.01). (**I**) Representative traces of KATP channel currents recorded from isolated ventricular myocytes in cell attached mode in the presence of the KATP channel opener DNP with and without H_2_O_2_. The vertical scale bar 5 pA, and the horizontal scale bar 2 s. (**J**) Number of KATP channel openings in each membrane patch under conditions with and without H_2_O_2_, as in Fig. 2D. One way ANOVA and Tukey’s multiple comparisons test were used for intergroup comparisons (*p < 0.05, **p < 0.01). (**K**) Summary data of open probability (NPo) in KATP channels analyzed from cell membrane patches shown in (**J**). One way ANOVA was used for comparison between all groups (P > 0.05). WT (4–7 cells, 2 mice), T224A (13–23 cells, 4 mice). (**L**) Expression of Kir6.2 in the heart was measured by RT-qPCR (Normalized against Gapdh, n = 5 for each genotype).

The results up to this point showed that ox-CaMKIIδ was important for reducing KATP channel activity and cell membrane expression. We next turned our attention to threonine 224 on Kir6.2 (T224), the pore forming subunit of KATP, because T224 phosphorylation by CaMKII decreases I_KATP_ by facilitating KATP sequestration from the sarcolemma^18,19^. We used CRISPR/Cas9 technology (see Methods and Supplementary Fig. 3A) to generate a new mouse model with knock-in replacement of T224 with alanine (T224A) (Fig. 3A,B). T224A mice were viable and born in predicted Mendelian ratios. Despite the known role of KATP in insulin secretion^30^, T224A mice had normal insulin secretion responses (Fig. 3C–E) and glucose (Fig. 3F) tolerance test results similar to WT controls. Furthermore, T224A and WT littermate control mice demonstrated similar exercise capacity on a treadmill running test (Supplementary Fig. 3B,C), maximum exercise stimulated O_2_ uptake (Supplementary Fig. 3D), and RER (Supplementary Fig. 3E). As predicted based on the demonstrated role of T224 in CaMKII-mediated KATP sequestration^18,19^, and on our findings that ox-CaMKII was essential for loss of sarcolemmal KATP channel expression (Fig. 2), we found that I_KATP_ density was reduced by H_2_O_2_ (0.4 mM) in cardiomyocytes isolated from WT (Fig. 3G left panel and Fig. 3H left two bars), but not from T224A mice (Fig. 3G right panel and Fig. 3H right two bars). We elicited I_KATP_ using the same ramp voltage command as in Fig. 2A inset, and applied pinacidil (0.1 mM) and 2, 4 dinitrophenol (DNP, 0.1 mM) to induce I_KATP_ (red lines in Fig. 3G for both WT and T224A representative traces), and glibenclamide (3–6 µM) to eliminate I_KATP_ (blue lines in Fig. 3G for both WT and T224A representative traces). We found that pretreatment of H_2_O_2_ significantly reduced I_KATP_ density in ventricular myocytes isolated from WT mice (black line in the left panel of Fig. 3G and summary data in Fig. 3H), but that H_2_O_2_ application had almost no effect on I_KATP_ density in T224A ventricular myocytes (black line in the right panel of Fig. 3G and summary data in Fig. 3H). We confirmed similar findings in cell-attached mode patch clamp recordings (Fig. 3I–K), where the number of KATP channels in each cell membrane patch and KATP channel Po were not affected by H_2_O_2_ treatment in T224A myocytes. However, basal I_KATP_, in the presence of pinacidil and DNP (see Fig. 2A), was reduced compared to I_KATP_ density measured in WT and MMVV ventricular myocytes (Fig. 3G right panel and Fig. 3H right two bars). Despite the lower density of KATP in T224A ventricular myocytes, there was no change in mRNA (Fig. 3L). We interpreted these data to suggest that constitutive knock-in replacement of T224 protected against ROS induced loss of KATP from sarcolemma, but unexpectedly led to a reduction in basal sarcolemmal KATP expression.

### Pinacidil protects against cell death after I/R injury in WT but not MMVV ventricular myocytes

**Figure 4:**
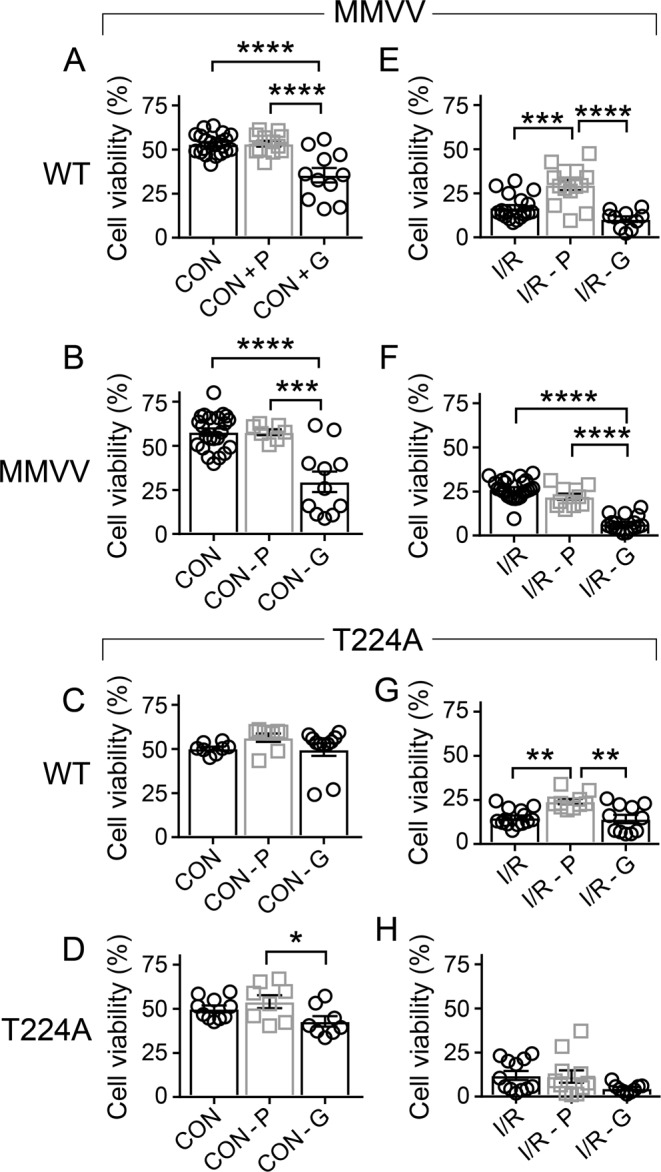
Ox-CaMKII resistant MMVV and pinacidil provide non-additive protection against I/R injury in isolated ventricular myocytes. (**A**–**D**) Summary data for cell viability in ventricular myocytes isolated from WT littermates of MMVV mice (**A**, cells from 3–9 mice/group), MMVV mice (**B**, cells from 4–10 mice/group), WT littermate of T224A mice (**C**, cells from 3 WT mice) and T224A mice (**D**, cells from 4 T224A mice) under control conditions (−oil) without drug treatment (left bar), with pinacidil 10 µM (middle bar), or with glibenclamide 2 µM (right bar). One way ANOVA was used for statistical analysis; Tukey’s multiple comparisons test was used for intergroup comparisons (*p < 0.05, ***p < 0.001, ****p < 0.0001). (**E**–**H**) Summary data for viability of ventricular myocytes isolated from WT littermate of MMVV mice (**E**, cells from 3–9 mice/group), MMVV mice (**F**, cells from 4–10 mice/group), WT littermates of T224A mice (**G**, cells from 3 WT mice) and T224A mice (**H**, cells from 4 T224A mice) under simulated I/R condition (+oil) without drug treatment (left bar), with pinacidil 10 µM (middle bar), or with glibenclamide 2 µM (right bar). One way ANOVA was used for statistical analysis; Tukey’s multiple comparisons test was used for intergroup comparisons (**p < 0.01, ***p < 0.001, ****p < 0.0001).

**Figure 5:**
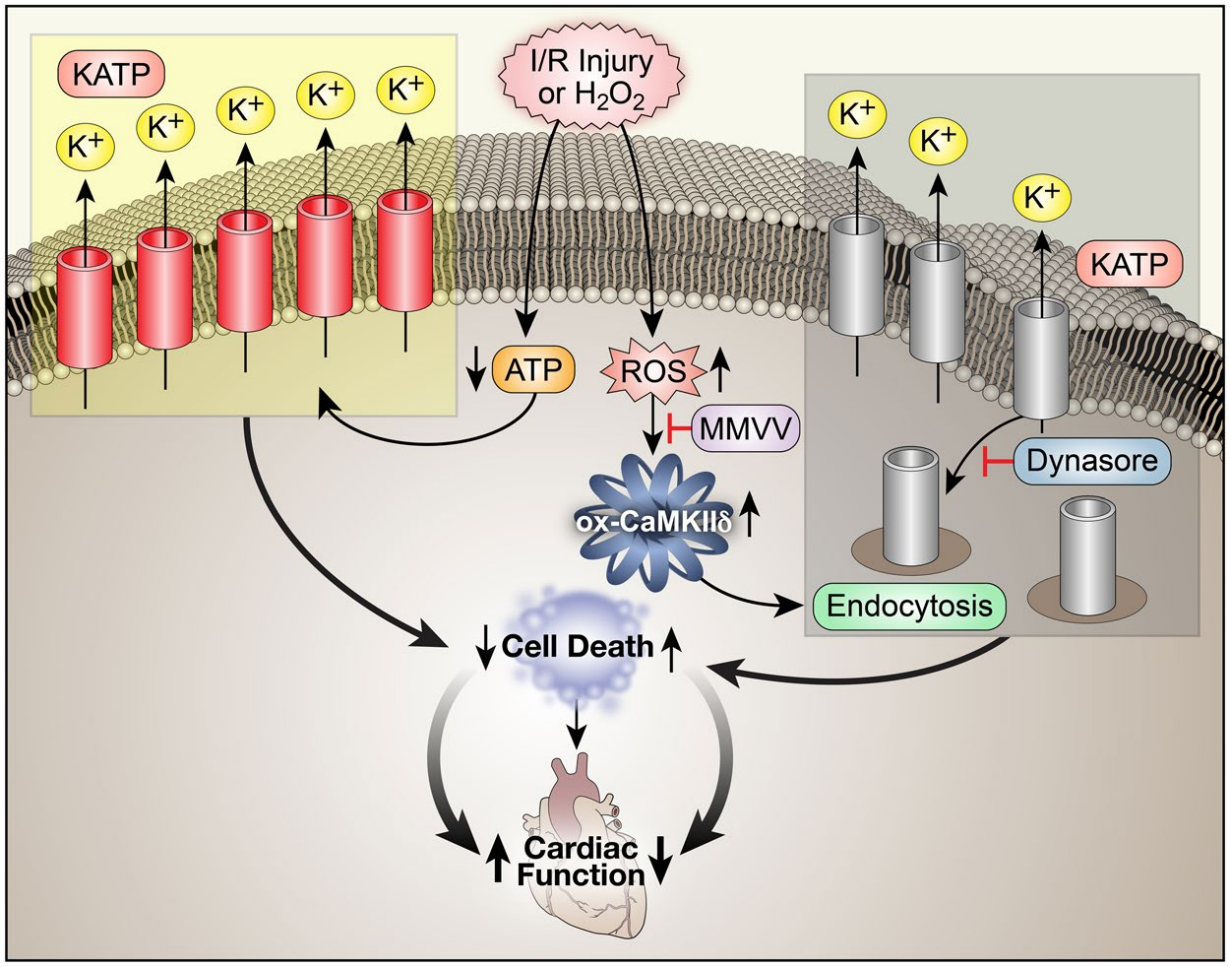
The proposed pathway for ox-CaMKII causing increased myocardial death after I/R injury by enhancing sequestration of KATP channels. I/R injury leads to reduced ATP and increased ROS. Reduced ATP increases KATP opening to protect cardiomyocytes, but ROS oxidize CaMKII, and ox-CaMKII enhances KATP endocytosis leading to reduced I_KATP_ and increased myocardial death. Mice lacking ox-CaMKIIδ (MMVV) are relatively protected from loss of I_KATP_ and myocardial death after I/R. Dynasore, an endocytosis blocker added to WT ventricular myocytes, and ventricular myocytes isolated from MMVV mice show similar protection against loss of I_KATP_.

We next compared survival, before and after I/R injury, in isolated ventricular myocytes at baseline with pinacidil, or glibenclamide (Fig. 4). Pinacidil is a KATP agonist (i.e. ‘opener’) while glibenclamide is a KATP antagonist. Pinacidil is known to improve while glibenclamide worsens myocardial survival in response to I/R injury^31,32^. In order to test if benefits of KATP activation added to the increased survival in the MMVV cardiomyocytes after I/R injury, we next measured cardiomyocyte viability in response to I/R injury in the presence of pinacidil (10 μM). Pinacidil did not significantly affect cell survival in any of the groups under mock I/R control conditions (Fig. 4A–D, left panels, middle data set). However, pinacidil significantly increased survival in ventricular myocytes isolated from WT mice after I/R injury (Fig. 4E,G, compare middle and left data sets in each panel). Pinacidil did not significantly change survival after I/R injury in MMVV (Fig. 4F) or T224A ventricular myocytes (Fig. 4H). These data suggested that MMVV improved survival, at least in part, by increasing I_KATP_ because the benefits of MMVV and pinacidil were not additive. Glibenclamide (2–4 μM), a KATP antagonist, tended to reduce the viability of isolated ventricular myocytes compared to pinacidil treated groups in the absence (Fig. 4A–D) or presence (Fig. 4E–H) of I/R injury (compare left and right data sets in each panel). Taken together, these data supported a concept where ox-CaMKII reduces I_KATP_ leading to cardiomyocyte death after I/R injury (Fig. 5).

## Discussion

The role of ROS in promoting I/R injury is widely accepted^33^. However, the complexity of ROS mediated injury presents a major barrier to unambiguous, and precise identification of downstream targets affected by ROS that contribute to pathological consequences of I/R injury. CaMKII has emerged as a ROS-activated signal with the potential to activate pro-death pathways in myocardium in response to myocardial infarction, angiotensin II^7^, and aldosterone^34^. The MMVV CaMKII mutant is resistant to ROS-triggered activation, but retains other wild type attributes, including activation by calcified calmodulin and the capacity to demonstrate Ca^2+^ and calmodulin independent activity by threonine 287 autophosphorylation^7^. Thus, the MMVV knock-in mouse model represents an important tool to test for a potential contribution of ox-CaMKII to I/R mediated injury. The protection response of MMVV mice to I/R injury provides new, direct evidence that ox-CaMKII is a critical transduction element, coupling ROS to cardiomyocyte death and myocardial dysfunction. Our data also confirm that ox-CaMKII couples ROS to KATP, and that under pathological conditions ox-CaMKII contributes to cardiomyocyte death, at least in part, by reducing I_KATP_.

I/R injury is a pathological insult that activates diverse ‘upstream’ signals, including increased ROS, with the potential to contribute to myocardial injury, dysfunction, and death^35^. The MMVV mice are incompletely protected from myocardial death and dysfunction in our study, showing that other, ox-CaMKII independent, pathways contribute to myocardial responses to I/R injury. CaMKIIδ is enriched in myocardium, but is also widely expressed in other tissues so ox-CaMKIIδ may contribute to I/R injury by extramyocardial actions, including by activation of inflammatory signaling cascades^36^. Thus, we do not interpret our results to rule out the possibility that ox-CaMKII contributes to I/R survival responses by actions outside of cardiomyocytes. However, given the complexity of I/R injury, the apparent magnitude of the ox-CaMKII contribution to I/R injury is remarkable, but nevertheless consistent with other findings showing MMVV mice resist complex, ROS-associated, triggers for myocardial disease. MMVV knock-in mice are known to harbor disease resistance to alcoholic cardiomyopathy^37^, cardiomyopathy in Duchenne’s muscular dystrophy^38^, diabetic cardiomyopathy^13^, and angiotensin II-primed atrial fibrillation^14^. The MMVV cardiomyocytes showed a similar resistance to H_2_O_2_ and I/R injury, suggesting the component of I/R injury protected by the MMVV mutation was related to ROS. The MMVV knock-in mutation was made on the CaMKIIδ background, leaving CaMKIIγ, a minor myocardial isoform capable of generating ox-CaMKII. The resistance of MMVV mice to I/R injury appears to confirm earlier findings in CaMKIIδ knock-out mice^39–41^, indicating that CaMKIIδ is the major CaMKII isoform contributing to disease in mouse models.

CaMKII is a multifunctional serine threonine kinase with a diverse array of molecular substrates. Regulation of intracellular Ca^2+^ is a major focus for CaMKII actions in the cytoplasmic compartment. CaMKII catalyzed phosphorylation of ryanodine receptors^42,43^, voltage-gated Ca^2+^ ^44,45^, and Na^+^ ^46^ channels all contribute to increasing cytoplasmic Ca^2+^. CaMKII actions at KATP can likely be considered within the framework of augmenting cytoplasmic Ca^2+^ concentration because reduced I_KATP_ has the potential to prolong action potential repolarization, and thereby promote cellular Ca^2+^ entry^15^. We speculate that CaMKII mediated increases in intracellular Ca^2+^ contribute to myocardial performance under physiological stress, but become maladaptive during pathological stress, leading to Ca^2+^ overload and myocardial death.

KATP channels are mostly closed under normal physiological conditions, but KATP channels open under ischemic conditions in response to a local reduction in ATP. There are at least 3 mechanisms by which opening of KATP protects cardiac myocytes from injury and death^15^: 1. Increased K^+^ conductance stabilizes the resting membrane potential, preventing triggered depolarizations that could augment Ca^2+^ channel opening; 2. I_KATP_ shortens the action potential plateau, resulting in reduced voltage gated Ca^2+^ channel current; 3. KATP opening conserves intracellular energy stores by reducing intracellular Ca^2+^and contraction. Our cellular I/R experimental findings are consistent with *in vivo*^47,48^ and *in vitro*^49–51^ studies demonstrating that KATP channel opening protects against ischemic myocyte death.

MMVV and WT Dynasore treated ventricular myocytes were protected against loss of I_KATP_ density after H_2_O_2_, consistent with previous reports that Ca^2+^ activation of CaMKII triggered dynamin-dependent internalization of KATP channels. This process required phosphorylation of threonine at 180 and 224 and an intact (330)YSKF(333) endocytosis motif of the KATP channel Kir6.2 pore-forming subunit^18^. In pancreatic β cells, CaMKII-dependent phosphorylation at Kir6.2 T224 reduces KATP cell membrane expression, and is enabled by β_IV_-spectrin targeting^19^. Loss of KATP in skeletal muscle caused mice to be energetically inefficient, lean, and exhibit poor exercise tolerance^52^. These and other studies^27^, suggested to us that the T224A mutation, which biased against dynamic changes in KATP, should have broad-ranging consequences. However, despite these considerations, the T224A mice showed normal glucose-stimulated insulin secretion, normal glucose, and exercise tolerance similar to WT littermate controls. Isolated cardiomyocytes from T224A mice had reduced basal I_KATP_, and were not protected from I/R injury or H_2_O_2_. We interpret these data to suggest either that congenital absence of Kir6.2 T224 results in yet unknown compensatory changes, and/or that CaMKII actions at KATP involve multiple sites.

I/R injury is a clinically important issue^4,53^. However, I/R models are notoriously misleading, and in some cases have provided proof of concept evidence for various pathways manipulated to reduce myocardial death that ultimately failed to provide anticipated benefits in clinical trials^53–55^. Here we used an *in vivo* I/R model, as a starting point, to avoid many potential pitfalls of less reliable, and potentially less clinically relevant *in vitro* and *ex vivo* studies^56^. We also focused on a highly validated pathway for concisely regulating ROS induced tissue injury by replacing CaMKIIδ regulatory domain methionines with valines. Thus, our finding that ox-CaMKII is required for a substantial amount of myocardial death after I/R injury adds to evidence that ox-CaMKII plays an important role in myocardial survival. Because of the consistently disappointing results of clinical trials of anti-oxidant agents for cardiovascular disease^57^, and the apparent protection exhibited by MMVV mice we hypothesize that therapeutic modulation of ROS will require precision targeting of pathological signals downstream to ROS. Ox-CaMKII may be such a signal, but to our knowledge, there have been no clinical trials with a CaMKII inhibitor drug, so our findings await translational testing in patients.

## Methods

### Animal use

All animal handling procedures were in accordance with National Institutes of Health guidelines and were approved by the Institutional Animal Care and Use Committees of Johns Hopkins University School of Medicine. Two kinds of Knock-in mice: MMVV and T224A were used in this study. The MMVV mice have been described^13,14^. We developed the T224A mice for this study. All comparisons between knock-in MMVV and T224A and WT used WT littermate controls. Based on our preliminary experimental findings, and documented gender differences in KATP^58^, we used only male mice in this study.

### Generation of Kir6.2-T224A point mutation in mice by CRISPR-mediated homologous recombination

See detail in Supplementary Materials.

### Glucose tolerance test

The mice fasted for 16 hours (overnight). 20% glucose (w/v in normal saline) was injected into the peritoneum at 2 g/kg. Glucose in tail blood was measured before the injection, as well as 15, 30, 60, 90, 120, and 180 minutes after the injection with a OneTouch Ultra 2 blood glucose meter (Lifescan Inc., Milpitas, CA).

### Glucose-induced insulin secretion

To measure glucose-induced insulin section, mice were fasted and injected with glucose solution in the same manner as for the glucose tolerance test. Tail blood was collected before and 15 minutes after glucose injection. Serum insulin was measured with an ultra-sensitive mouse insulin ELISA kit (Crystal Chem Inc, Elk Grove Village, IL, Catalog # 90080).

### Exercise capacity and metabolic treadmill

See detail in Supplementary Materials.

### Isolation of cardiac total RNA

To isolate total RNA, 10 mg tissue from the ventricular apex region were homogenized in the Trizol Reagent (ThermoFisher Scientific, Waltham, MA, catalog #15596026). The aqueous phase from the chloroform extraction was processed by the RNeasy Mini Kit (Qiagen, Hilden, Germany · Venlo, Netherlands, catalog #74104) with on-column DNase treatment to eliminate genomic DNA contamination.

### RT-qPCR

60 ng of total RNA were converted into cDNA with the iScript™ Reverse Transcription Supermix (Bio-Rad, Hercules, CA catalog # 1708840). 2 ng of cDNA were used in each qPCR reaction on a CFX Connect Real-time PCR detection system (Bio-Rad, Hercules, CA) with SsoAdvanced™ Universal SYBR® Green Supermix (Bio-Rad, Hercules, CA catalog # 1725271). The primers are pre-validated primePCR primers (Kcnj11: qMmuCED0001332 and Gapdh: MmuCED0027497). qPCR data were analyzed by the software Bio-Rad CFX Manager 3.1, using Gapdh expression as the loading control.

### ***In vivo*** I/R surgery

Myocardial I/R was performed *in vivo*^59^ by 45 min of ligation of the left anterior descending coronary artery followed by 24 h of reperfusion. Mice were anesthetized with 4% isoflurane for ~2 min and maintained at 1.5–2% isoflurane. Anesthetized mice were intubated and mechanically ventilated at 120 breaths per minute with a tidal volume of 200 µl. Mice are given buprenorphine (0.03–0.07 mg/kg) or Rimadyl (4–5 mg/kg) subcutaneously for post-operative analgesia. Through a sterile incision, a left thoracotomy and pericardiotomy was performed in the 5^th^ to 6^th^ intercostal space. The mice underwent coronary ligation using 7-0 prolene and PE10 tubing as a splint. After 45 minutes ischemia, the splint was removed and the suture released to reperfuse the heart. The chest and skin were closed with 5-0 silk suture. Following I/R, and prior to sacrifice, 1% solution of Evans Blue was injected into the cardiac apex to determine the area at risk or *in vitro* Langendorff perfusion of the aorta was used to perfuse Evans Blue^59,60^. Hearts were harvested and 1-mm sections of the hearts were stained with 1% triphenyl tetrazolium chloride (TTC) to measure the area of necrosis. The area at risk, area of necrosis, and left ventricular area were stored as digital images, and analyzed using ImageJ software.

### Simulated cellular I/R injury

Simulated ischemia was induced by layering mineral oil (0.5 ml for 40 min) over a thin film of media covering the cells followed by 45 min of ‘reperfusion’ in normal media in a 1.5 ml tube, as described^26^. Ventricular myocytes were enriched in a 25 µl pellet by gently centrifuging the cell suspension of 1.5 ml (30 s, 100 g). After removing the supernatant, we added 0.5 ml mineral oil to the tube to limit atmospheric diffusion to the cells. A control group was treated to the same centrifugation protocol and supernatant removal, but 0.5 ml of Tyrode’s solution was replaced without addition of oil. After 40 min the oil was removed and the cells were suspended in Tyrode’s solution (mM: NaCl 137, KCl 5.4, CaCl_2_ 1.2, MgCl_2_ 2, NaH_2_PO_4_ 0.33, Glucose 10, HEPES-NaOH 10, pH 7.4) for 45 min. The entire process was conducted in a 37 °C water bath.

### Cell viability measurements

Dissociated ventricular myocytes were stored in Tyrode’s solution at room temperature or at 37 °C, as indicated. We used morphology and Trypan blue (Corning or Gibco) staining to analyze cell viability, using 100 µl of cell suspension and 20 µl 0.4% Trypan blue for 5 min. The number of stained and unstained (round or square) cells were counted as dead cells; viable cells were counted as cells with a rod-shaped morphology and a length/width ratio of more than 3. Cell viability was expressed as the percentage of viable cells amongst total cells. The total count (for one sample point) ranged between 200 ->1000 cells (from counting at least 7 fields of view), and was completed within 15 min to preclude nonspecific uptake of Trypan blue. Cell imaging was performed with a Nikon Eclipse Ti inverted phase contrast microscope at 100X magnification. The images were stored in a computer and cell counting was performed by NIS Elements imaging software (Nikon) and validated by visual inspection of each image.

### I_KATP_ whole cell mode and KATP single channel recording

Isolated ventricular myocytes were placed in a recording chamber (RC-26, Warner Instruments, Hamden, CT, USA) on the stage of an inverted microscope (Nikon ECLIPSE-Ti). Myocytes were continuously perfused with modified Tyrode’s solution (mM: NaCl 137, KCl 5.4, CaCl_2_ 1, MgCl_2_ 2, NaH_2_PO_4_ 0.33, HEPES-NaOH 10, pH 7.4). Whole cell membrane current was recorded at room temperature using Axopatch-200B amplifier (Molecular Devices, Sunnyvale, CA, USA). Patch pipettes were made using borosilicate glass (OD 1.5 mm) and had resistances between 4 and 6 MΩ when filled with this solution (mM: KCl 140, KATP 0.1, KADP 0.1, MgCl_2_ 1, HEPES 10, and EGTA 1, titrated to pH 7.2 with KOH). Whole-cell current was recorded at a holding potential of −45 mV. Current–voltage relationships were obtained using a ramp protocol (5 to −100 mV at 25 mV/S, applied every 20 s). Membrane currents were filtered (low-pass Bessel response with a cut-off frequency of –3 dB at 1–2 kHz), digitized at 5 kHz, and stored on a computer hard disk with pCLAMP software (Clampex 10.07, Molecular Devices, Sunnyvale, CA, USA).

Single channel KATP currents were mostly recorded in cell-attached mode using a recording chamber (RC26, Warner, Hamden, CT, USA), bath solution (mM: KCl 150; EGTA 5; HEPES, 10; pH adjusted to 7.2 with KOH), and pipette solution (mM: KCl 150; CaCl_2_, 2; and HEPES 10; pH adjusted to 7.2 with KOH). The use of symmetrical recording solutions (150 mM K^+^) resulted in an equilibrium potential for potassium (EK) and a resting membrane potential (Vm) around 0 mV, as determined from the I–V relationship of the KATP channel. All recordings were carried out at room temperature, and all patches were voltage clamped at −60 mV (i.e. with +60 mV pipette potentials). Single-channel currents were recorded with an Axopatch 200B patch-clamp amplifier (Molecular Devices, Sunnyvale, CA, USA), low-pass filtered (3 dB, 2 kHz) and digitized at 20 kHz online using Clampex 10.07 software (Molecular Devices, Sunnyvale, CA, USA) via a 16 bit A/D converter (Digidata 1440 A; Molecular Devices, Sunnyvale, CA, USA). Some single channel recordings were performed in excised cell membrane patches in the same solutions and conditions described for cell-attached mode recordings.

### Echocardiography

*In vivo* cardiac morphology was assessed by trans-thoracic echocardiography (Vevo 2100, 40 MHz transducer; VisualSonics Inc, Toronto, Canada) in conscious mice. As previously described^61^, the M-mode echocardiogram was acquired from the parasternal long axes view of the left ventricle at the mid-papillary muscles level and at sweep speed of 200 mm/sec. The end-diastolic and end-systolic ventricular volumes (EDV, ESV), were obtained from the two chamber view of the heart in long axis view, using Simpson’s method. The stroke volume (SV), and the percent ejection fraction (EF) were automatically calculated by the Vevo 2100 ultrasound system built in software. The studies and analysis were performed by an operator blinded as to the experimental group.

### Data analysis and statistics

Comparisons of multiple groups were performed using one way ANOVA followed by Tukey’s post-hoc correction or Sidak’s multiple comparisons test (GraphPad Prism). Two-group analysis used an unpaired 2-tailed Student’s *t* test. Statistically significant differences (defined as P ≤ 0.05) between genotypes (WT vs. MMVV or T224A) and respective treatment groups are indicated.

## Supplementary information

Supplementary materials

## Data Availability

The authors declare that the data supporting the findings of this study are available within the paper, Supplementary Material and from the authors on request.

## References

[1] Nabel EG, Braunwald EA (2012). Tale of coronary artery disease and myocardial infarction. N. Engl. J. Med..

[2] Ibáñez B, Heusch G, Ovize M, Van de Werf F (2015). Evolving therapies for myocardial ischemia/reperfusion injury. J. Am. Coll. Cardiol..

[3] Fuster V (2014). Top 10 cardiovascular therapies and interventions for the next decade. Nat. Rev. Cardiol..

[4] Yellon DM, Hausenloy DJ (2007). Myocardial reperfusion injury. N. Engl. J. Med..

[5] Chouchani ET (2014). Ischaemic accumulation of succinate controls reperfusion injury through mitochondrial ROS. Nature..

[6] Murphy MP (2014). Antioxidants as therapies: can we improve on nature?. Free Radic. Biol. Med..

[7] Erickson JR (2008). A dynamic pathway for calcium-independent activation of CaMKII by methionine oxidation. Cell..

[8] Palomeque J (2008). CaMKII mediates angiotensin II-induced cardiomyocytes apoptosis: role of Ca2+, ROS and p38 MAPK. J. Mol. Cell Cardiol..

[9] Howe CJ, Lahair MM, McCubrey JA, Franklin RA (2004). Redox regulation of the calcium/calmodulin-dependent protein kinases. J. Biol. Chem..

[10] Rellos P (2010). Structure of the CaMKIIδ/Calmodulin Complex Reveals the Molecular Mechanism of CaMKII Kinase Activation. PLoS Biol..

[11] Mattiazzi A (2015). Chasing cardiac physiology and pathology down the CaMKII cascade. Am. J. Physiol. Heart Circ. Physiol..

[12] Bell JR, Erickson JR, Delbridge LM (2014). Ca(2+)/calmodulin dependent kinase II: a critical mediator in determining reperfusion outcomes in the heart?. Clin. Exp. Pharmacol. Physiol..

[13] Luo M (2013). Diabetes increases mortality after myocardial infarction by oxidizing CaMKII. J. Clin. Invest..

[14] Purohit A (2013). Oxidized Ca(2+)/calmodulin-dependent protein kinase II triggers atrial fibrillation. Circulation..

[15] Foster MN, Coetzee WA (2016). KATP Channels in the Cardiovascular System. Physiol. Rev..

[16] Storey NM, Stratton RC, Rainbow RD, Standen NB, Lodwick D (2013). Kir6.2 limits Ca(2+) overload and mitochondrial oscillations of ventricular myocytes in response to metabolic stress. Am. J. Physiol. Heart Circ. Physiol..

[17] Li J (2007). Calmodulin kinase II inhibition enhances ischemic preconditioning by augmenting ATP-sensitive K+ current. Channels (Austin)..

[18] Sierra A (2013). Regulation of cardiac ATP-sensitive potassium channel surface expression by calcium/calmodulin-dependent protein kinase II. J. Biol. Chem..

[19] Kline CF (2013). IV-Spectrin and CaMKII facilitate Kir6.2 regulation in pancreatic beta cells. Proc. Natl. Acad. Sci. USA.

[20] Vila-Petroff M (2007). CaMKII inhibition protects against necrosis and apoptosis in irreversible ischemia-reperfusion injury. Cardiovasc. Res..

[21] Di Carlo MN (2014). CaMKII-dependent phosphorylation of cardiac ryanodine receptors regulates cell death in cardiac ischemia/reperfusion injury. J. Mol. Cell Cardiol..

[22] Gray CB (2017). CaMKIIδ subtypes differentially regulate infarct formation following *ex vivo* myocardial ischemia/reperfusion through NF-κB and TNF-α. J. Mol. Cell Cardiol..

[23] Qu J (2017). Oxidized CaMKII promotes asthma through the activation of mast cells. JCI Insight..

[24] Wang Q, Huang L, Yue J (2017). Oxidative stress activates the TRPM2-Ca2+/CaMKII-ROS signaling loop to induce cell death in cancer cells. Biochim. Biophys. Acta..

[25] Hoch B, Meyer R, Hetzer R, Krause EG, Karczewski P (1999). Identification and Expression of δ-Isoforms of the Multifunctional Ca2+/Calmodulin-Dependent Protein Kinase in Failing and Nonfailing Human Myocardium. Circ. Res..

[26] Diaz RJ, Wilson GJ (2006). Studying ischemic preconditioning in isolated cardiomyocyte models. Cardiovasc. Res..

[27] Yang HQ (2016). Elasticity of sarcolemmal KATP channel surface expression: relevance during ischemia and ischemic preconditioning. Am. J. Physiol. Heart Circ. Physiol..

[28] Sasaki N, Sato T, Marbán E, O’Rourke B (2001). ATP consumption by uncoupled mitochondria activates sarcolemmal K(ATP) channels in cardiac myocytes. Am. J. Physiol. Heart Circ. Physiol..

[29] Findlay I (1992). Inhibition of ATP-sensitive K+ channels in cardiac muscle by the sulphonylurea drug glibenclamide. J. Pharmacol. Exp. Ther..

[30] Ashcroft FM, Rorsman P (2013). K(ATP) channels and islet hormone secretion: new insights and controversies. Nat. Rev. Endocrinol..

[31] Cole WC, McPherson CD, Sontag D (1991). ATP-regulated K+ channels protect the myocardium against ischemia/reperfusion damage. Circ. Res..

[32] Shigematsu S (1995). Pharmacological evidence for the persistent activation of ATP-sensitive K+ channels in early phase of reperfusion and its protective role against myocardial stunning. Circulation..

[33] Becker LB (2004). New concepts in reactive oxygen species and cardiovascular reperfusion physiology. Cardiovasc. Res..

[34] He BJ (2011). Oxidation of CaMKII determines the cardiotoxic effects of aldosterone. Nat. Med..

[35] Murphy E, Steenbergen C (2008). Mechanisms underlying acute protection from cardiac ischemia-reperfusion injury. Physiol. Rev..

[36] Weinreuter M (2014). CaM Kinase II mediates maladaptive post-infarct remodeling and pro-inflammatory chemoattractant signaling but not acute myocardial ischemia/reperfusion injury. EMBO Mol. Med..

[37] Mustroph J (2018). SR Ca2+-leak and disordered excitation-contraction coupling as the basis for arrhythmogenic and negative inotropic effects of acute ethanol exposure. J. Mol. Cell Cardiol..

[38] Wang Q (2018). Oxidized CaMKII (Ca2+/Calmodulin-Dependent Protein Kinase II) Is Essential for Ventricular Arrhythmia in a Mouse Model of Duchenne Muscular Dystrophy. Circulation: Arrhythmia and Electrophysiology..

[39] Ling H (2009). Requirement for Ca2+/calmodulin-dependent kinase II in the transition from pressure overload-induced cardiac hypertrophy to heart failure in mice. J. Clin. Invest..

[40] Ling H (2013). Ca2+/Calmodulin-dependent protein kinase II δ mediates myocardial ischemia/reperfusion injury through nuclear factor-κB. Circ. Res..

[41] Backs J (2009). The delta isoform of CaM kinase II is required for pathological cardiac hypertrophy and remodeling after pressure overload. Proc. Natl. Acad. Sci. USA.

[42] Rodriguez P, Bhogal MS, Colyer J (2003). Stoichiometric phosphorylation of cardiac ryanodine receptor on serine 2809 by calmodulin-dependent kinase II and protein kinase A. J. Biol. Chem..

[43] Wehrens XH, Lehnart SE, Reiken SR, Marks AR (2004). Ca2+/calmodulin-dependent protein kinase II phosphorylation regulates the cardiac ryanodine receptor. Circ. Res..

[44] Anderson ME, Braun AP, Schulman H, Premack BA (1994). Multifunctional Ca2+/calmodulin-dependent protein kinase mediates Ca(2+)-induced enhancement of the L-type Ca2+ current in rabbit ventricular myocytes. Circ. Res..

[45] Xiao RP, Cheng H, Lederer WJ, Suzuki T, Lakatta EG (1994). Dual regulation of Ca2+/calmodulin-dependent kinase II activity by membrane voltage and by calcium influx. Proc. Natl. Acad. Sci. USA.

[46] Wagner S (2006). Ca2+/calmodulin-dependent protein kinase II regulates cardiac Na+ channels. J. Clin. Invest..

[47] Yao Z, Gross GJ (1994). Effects of the KATP channel opener bimakalim on coronary blood flow, monophasic action potential duration, and infarct size in dogs. Circulation..

[48] Grover GJ (1994). Protective effects of ATP-sensitive potassium-channel openers in experimental myocardial ischemia. J. Cardiovasc. Pharmacol..

[49] Mitani A (1991). Effects of glibenclamide and nicorandil on cardiac function during ischemia and reperfusion in isolated perfused rat hearts. Am. J. Physiol..

[50] Critz SD, Liu GS, Chujo M, Downey JM (1997). Pinacidil but not nicorandil opens ATP-sensitive K+ channels and protects against simulated ischemia in rabbit myocytes. J. Mol. Cell Cardiol..

[51] Cavero I, Djellas Y, Guillon JM (1995). Ischemic myocardial cell protection conferred by the opening of ATP-sensitive potassium channels. Cardiovasc. Drugs Ther..

[52] Alekseev AE (2010). Sarcolemmal ATP-sensitive K(+) channels control energy expenditure determining body weight. Cell Metab..

[53] Heusch G (2013). Cardioprotection: chances and challenges of its translation to the clinic. Lancet..

[54] Heusch G (2017). Critical Issues for the Translation of Cardioprotection. Circ. Res..

[55] Hausenloy DJ, Yellon DM (2016). Ischaemic conditioning and reperfusion injury. Nat. Rev. Cardiol..

[56] Ytrehus K (2000). The ischemic heart–experimental models. Pharmacol. Res..

[57] Chen AF (2012). Free radical biology of the cardiovascular system. Clin. Sci. (Lond)..

[58] Ranki HJ, Budas GR, Crawford RM, Jovanović A (2001). Gender-specific difference in cardiac ATP-sensitive K(+) channels. J. Am. Coll. Cardiol..

[59] Michael LH (1995). Myocardial ischemia and reperfusion: a murine model. Am. J. Physiol..

[60] Bohl S (2009). Refined approach for quantification of *in vivo* ischemia-reperfusion injury in the mouse heart. Am. J. Physiol. Heart Circ. Physiol..

[61] Wei H (2012). Endothelial expression of hypoxia-inducible factor 1 protects the murine heart and aorta from pressure overload by suppression of TGF-beta signaling. Proc. Natl. Acad. Sci. USA.

