# Supplementary material for: Myocardial death and dysfunction after ischemia-reperfusion injury require CaMKIIδ oxidation
Source: Sci Rep. 2019 Jun 26;9:9291. doi: 10.1038/s41598-019-45743-6 (PMC6595001; doi:10.1038/s41598-019-45743-6)
Supplement: Supplementary file 1 — Supplementary materials [file 41598_2019_45743_MOESM1_ESM.pdf]

**Myocardial death and dysfunction after ischemia-reperfusion injury require CaMKIIδ oxidation**

Yuejin Wu, Qinchuan Wang, Ning Feng, Jonathan M. Granger, Mark E. Anderson*.

## Supplementary materials

## Supplementary Methods

### Generation of Kir6.2-T224A knock-in mice by CRISPR-mediated homologous recombination

The genomic sequence of *Kcnj11* gene encoding Kir6.2 (gi|372099103|ref|NC_000073.6|:c46099686-46098775, GRCm38.p4 C57BL/6J) was used to design the CRISPR guides and templates for homology-directed repair (HDR) with the web tool http://crispr.mit.edu/. The guide #8 (AACTTCGCCCTCGGGGCTGG) was chosen due to its close proximity to the codon for threonine 224 and lack of high probability off-targets (Supplementary Fig 3A). A single strand 150nt ultramer DNA oligo (Kcnj11-ssODN-g8) was designed as the template for HDR-mediated point mutations. The sequence of Kcnj11-ssODN-g8 is "CTGCGCGTAGGGGACCTCCGAAAGAGCATGATCATTAGCGCCACCATCCACATGCAGGTGGTGCGCAAGACTGCTAGCCCCGAGGGCGAAGTTGTGCCTCTCCACCAGGTAGACATCCCCATGGAGAATGGCGTGGGTGGTAACGGCATC". Both the HDR template and the single guide RNA (sgRNA) based on the sequence of guide #8 were ordered from Integrated DNA Technologies (IDT). CRISRP modified mice on the C57BL/6J background were generated by the Transgenic Mouse Core at Johns Hopkins University, School of Medicine. The founder mice were bred with C57BL/6J mice and the resulting F1 progeny was used for further breeding. 4 generations were bred prior to use.

For genotyping, genomic DNA was extracted from tail clips and a 341 bp fragment was amplified by PCR with primers TGCAGAATATCGTCGGGCTG, and GTTGGAGTCGATGACGTGGT. The PCR product was sequenced to detect the modification. The PCR product from the mutant allele was also detected through restriction enzyme digestion by NheI-HF or NheI (New England Biolabs, Ipswich, MA), resulting in 219bp and 122 bp fragments (Fig 3B).

### Exercise capacity and metabolic treadmill

Exercise capacity, oxygen consumption rate ($VO_2$), and respiratory exchange ratio (RER) were measured with a single lane modular metabolic treadmill for mice (Columbus Instrument,

Columbus, OH ) coupled with an Oxymax indirect calorimeter (Columbus Instrument, Columbus, OH). The Oxymax was calibrated with standard gas containing 0.50% $CO_2$ and 20.50% $O_2$ (Airgas Inc., Radnor, PA ) before each test. The inflow and outflow gas through the metabolic treadmill was sampled every 10 seconds by the Oxymax.

Prior to exercise the mice were acclimated to the treadmill for three sessions on three consecutive days. The treadmill was set to 10° inclination and the speed was increased from 0, 5, and 10 m/min during each acclimation session. The electric shock grid at the rear end of the treadmill was turned on and set at stimulation intensity of 9 and frequency of 1 shock per second.

During exercise capacity test, a single mouse was placed into the metabolic treadmill set at 10° inclination and stayed still for 20 minutes for the $VO_2$ reading to stabilize. The exercise protocol consisted of the following steps: (1) 10 m/min for 120s as warm up, (2) continuous acceleration from 15/min at a rate of 0.6 m/min$^2$ until the mouse was exhausted. Exhaustion was determined when the mouse stayed on the shock grid continuously for 5 seconds.

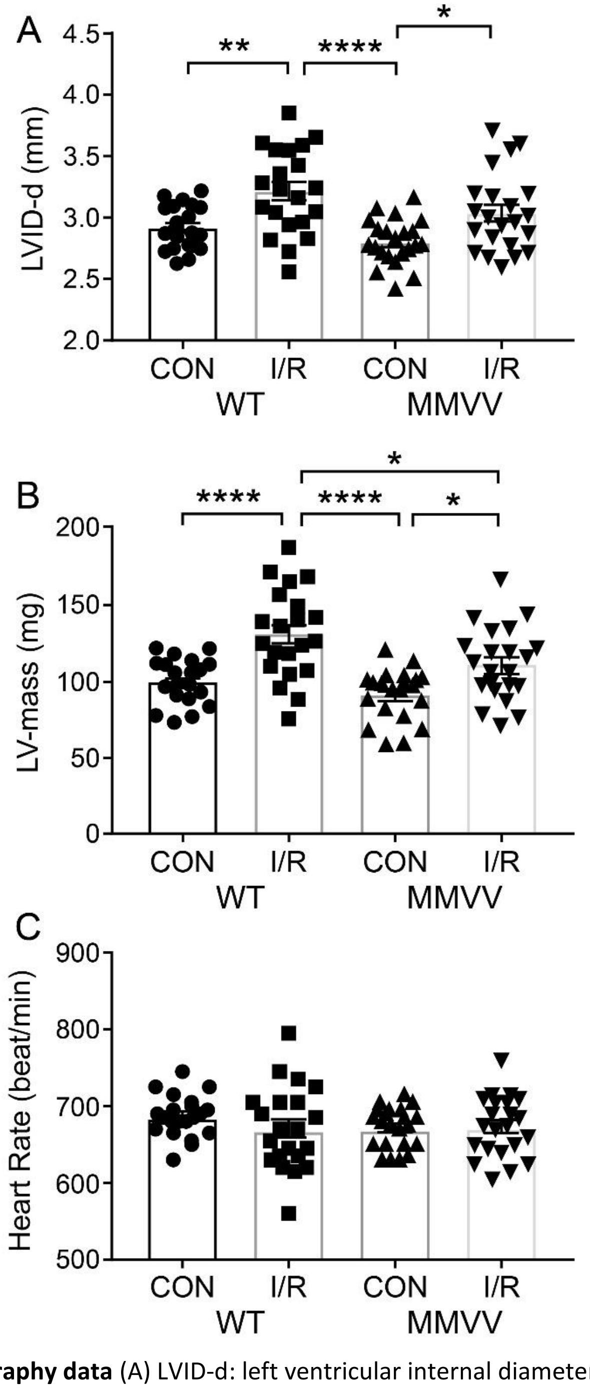


**Supplementary Fig. 1 Echocardiography data** (A) LVID-d: left ventricular internal diameter in diastole, (B) LV-mass, (C) Heart rate before (CON) and after I/R surgery in MMVV (n=22) and WT (n=21) mice. One way ANOVA was used for comparison between all groups (P<0.0001 for panel A and B); Tukey's multiple comparisons test was used to compare each group, as indicated by brackets *p<0.05, **p<0.01, ***p<0.001, ****p<0.0001.

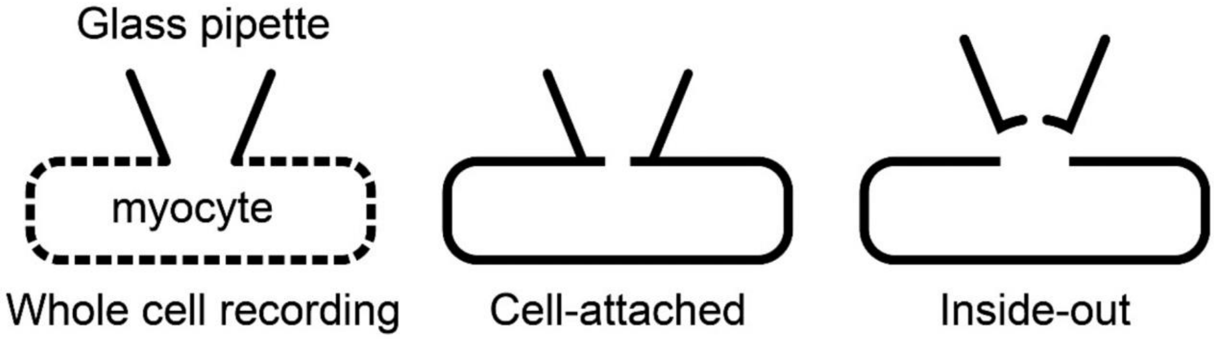


**Supplementary Fig. 2 Schematic diagrams for indicating patch clamp configurations.** Whole cell mode recordings were used to record macroscopic $I_{KATP}$, and cell-attached and excised inside-out configurations were used to record unitary KATP channel current. See Methods for detailed descriptions.

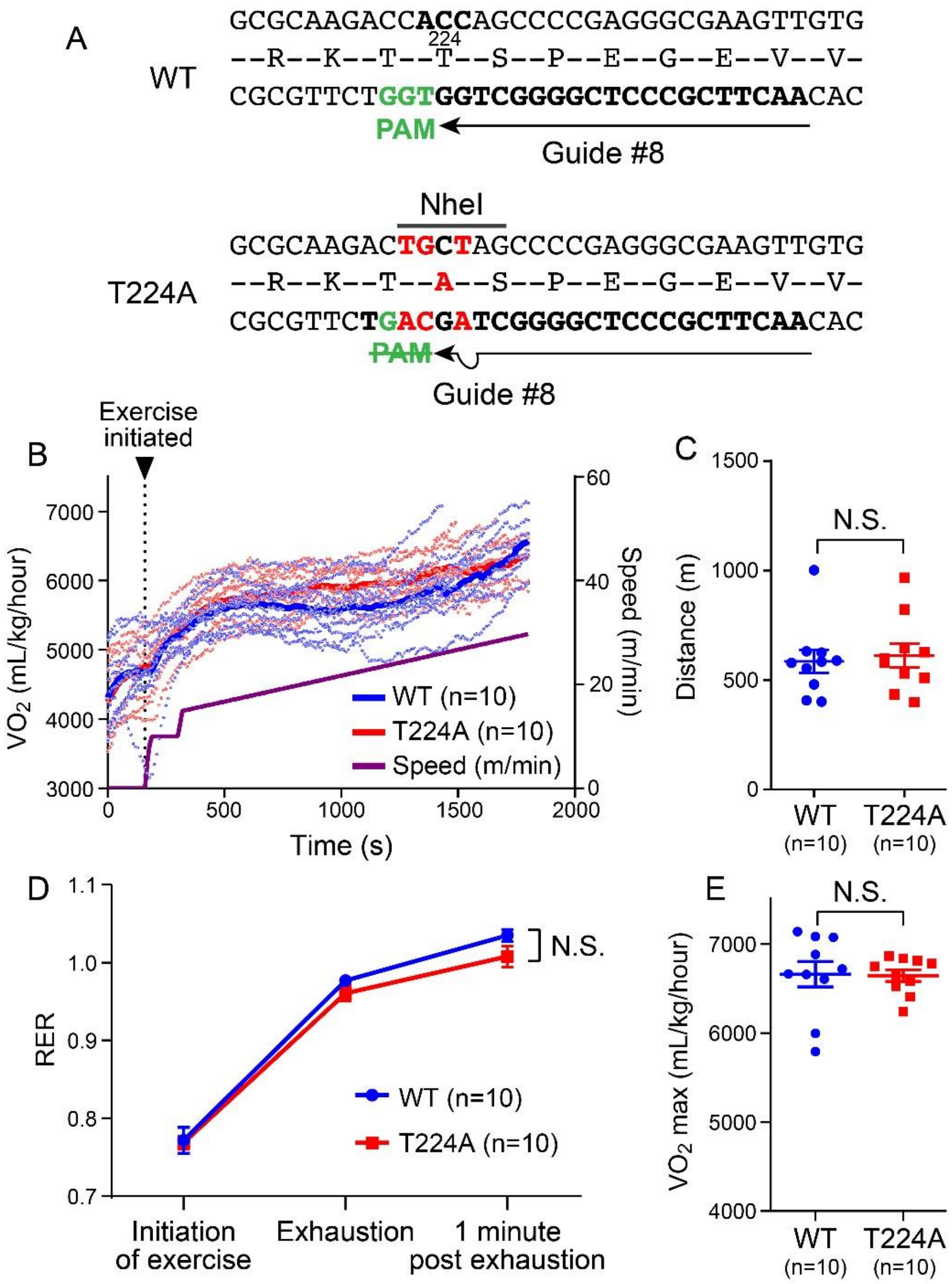


**Supplementary Fig. 3 T224A knock-in mice** (A) Genomic regions encoding amino acid T224 of the wildtype (*WT*) and A224 of the edited (*T224A*) alleles of *Kcnj11*. CRISPR guide #8 was used to target the wildtype allele. The homology directed repair mutates three nucleotides to generate T224A mutation, interrupt guide 8 binding, eliminate the PAM motif, and create a novel NheI restriction site. (B) Oxygen consumption rate ($VO_2$) during running exercise on a progressive ramp. Traces for individual mice were plotted semi-transparently and the mean values for each genotype were plotted as solid lines. Because sample sizes reduced when each mouse became exhausted, traces were truncated at 1810s for clarity. However, $VO_2$max data were derived from intact traces. The treadmill speed was plotted against time and shown on the right y-axis. (C) Treadmill exercise performance was similar between T224A and WT mice. (D) Respiratory exchange ratio (RER) at the moment of exercise initiation, exhaustion, and 1 minute after exhaustion. Running significantly increased RER (p<0.0001) but the genotype had

no effect (2-Way ANOVA). At the moment of exhaustion, the RER approached 1, suggesting that the running intensities were high and the mice relied almost exclusively on carbohydrate metabolism at this point. Immediately after exhaustion, the RER overshoot transiently and slightly exceeded 1.0. Thereafter, RER started to decrease (not shown). (E) No difference was detected in maximal oxygen consumption rate ($VO_2$ max) between *WT* and *T224A* mice.

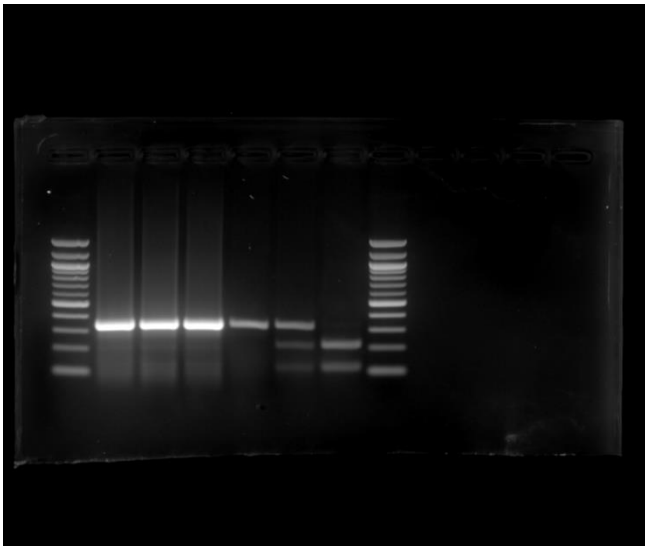

## **Original, uncropped file (Fig3B)**
